# Auxeticity of Yukawa Systems with Nanolayers in the (111) Crystallographic Plane

**DOI:** 10.3390/ma10111338

**Published:** 2017-11-22

**Authors:** Paweł M. Pigłowski, Jakub W. Narojczyk, Artur A. Poźniak, Krzysztof W. Wojciechowski, Konstantin V. Tretiakov

**Affiliations:** 1 Institute of Molecular Physics, Polish Academy of Sciences, Smoluchowskiego 17/19, 60-179 Poznan, Poland; pmp@ifmpan.poznan.pl (P.M.P.); kww@ifmpan.poznan.pl (K.W.W.); kvt@ifmpan.poznan.pl (K.V.T.); 2 Department of Technical Physics, Poznan University of Technology, Piotrowo 3, 60-695 Poznan, Poland; aap@man.poznan.pl

**Keywords:** auxetics, negative Poisson’s ratio, nanolayer, Yukawa potential, Monte Carlo simulations

## Abstract

Elastic properties of model crystalline systems, in which the particles interact via the hard potential (infinite when any particles overlap and zero otherwise) and the hard-core repulsive Yukawa interaction, were determined by Monte Carlo simulations. The influence of structural modifications, in the form of periodic nanolayers being perpendicular to the crystallographic axis [111], on auxetic properties of the crystal was investigated. It has been shown that the hard sphere nanolayers introduced into Yukawa crystals allow one to control the elastic properties of the system. It has been also found that the introduction of the Yukawa monolayers to the hard sphere crystal induces auxeticity in the $\left[11\bar{1}\right]\left[112\right]$-direction, while maintaining the negative Poisson’s ratio in the $\left[110\right]\left[1\bar{1}0\right]$-direction, thus expanding the partial auxeticity of the system to an additional important crystallographic direction.

## 1. Introduction

The colloidal crystals [1] have been a subject of intense studies in recent years [2,3,4,5,6,7,8,9]. This is mainly due to their potential applications [10,11,12]. Some physical properties of charge-stabilized colloids can be well described using the hard-core repulsive Yukawa potential [13,14,15,16,17,18]. Recently, it has been shown that systems with such kind of interaction exhibit interesting elastic properties, namely these systems reveal a negative Poisson’s ratio [19]. The Poisson’s ratio describes the lateral mechanical response under the longitudinal uniaxial strain of infinitesimal magnitude [20]. Materials with negative Poisson’s ratio (so-called *auxetics*) [21] increase their dimensions in directions perpendicular to the applied stretching and shrink in directions perpendicular to the applied compression. In general, Poisson’s ratio depends on both the direction of applied deformation and the direction of measurement of response to external stress. Materials in which the Poisson’s ratio is negative only in some crystallographic directions are called *partial auxetics* [22].

Auxetics are a relatively new class of materials. A few decades ago, the first man-made material with a negative Poisson’s ratio [23] and the model system [24] exhibiting auxetic properties were presented. Since then, one can observe quickly growing interest in this class of materials because of their unusual, counter-intuitive properties [25,26,27,28], which originate from various mechanisms involving both micro- and macroscopic structures [23,29,30,31,32,33,34,35,36,37,38], inter-particle interactions [24,39,40,41] as well as special conditions applied to the system [42,43]. At present, research is being conducted extensively in the pursuit for auxetic properties in already existing and new materials [39,44,45,46,47,48,49,50,51,52], the fabrication of auxetic composites [53,54,55], the construction of models for explaining auxetic properties [56,57,58,59,60,61,62,63,64], and the search for new mechanisms leading to the appearance of auxetic properties in various systems. The studies on the influence of structural modifications on the auxetic properties of Yukawa crystals are an example of the latter [65,66]. In the referenced studies, it has been shown that the introduction of nanochannel filled by hard spheres into Yukawa crystal enables enhancing the auxetic properties [65]. On the other hand, the introduction of the monolayer of hard spheres in the Yukawa crystal oriented in the (010) crystallographic plane leads to the appearance of a new auxetic direction to emerge [66]. Gathering the facts, the following question arises: what effect on the auxetic properties will the introduction of nanolayer into the Yukawa crystal oriented in a different crystallographic direction have?

This work concerns the study of the effects of structural modification in the form of insertion into crystal of nanolayers that are oriented parallel to the (111) crystallographic plane. The aim of this work is to determine the elastic properties of such structurally modified crystals, in which the particles interact via hard and hard-core repulsive Yukawa potentials. In particular, we examine the effect of this structural modification on the auxetic properties of the system under study. The purpose of this paper is to present data that show that the discussed structural modifications have a significant effect on auxeticity of the studied model system. We believe that in a not far future it will be possible to use this knowledge in designing real materials.

## 2. Model and Computational Details

In the considered model, the initial face-centered cubic structure forms by particles interacting through the hard-core repulsive Yukawa potential (HCRYP) [13,14,15]

(1) $$\beta u_{ij}=\left\{\begin{array}{cc} \infty , & r_{ij}<\sigma ,\\ \beta \varepsilon \frac{\exp[-\kappa \sigma \left(r_{ij}/\sigma -1\right)]}{r_{ij}/\sigma }, & r_{ij}\geq \sigma , \end{array}\right.$$

where $\beta =1/k_{B}T$, $k_{B}$ is the Boltzmann constant, *T* is the temperature, σ is the diameter of the particles’ hard core, ϵ is the contact potential, and κ is the inverse of the Debye screening length. In order to form a system with nanolayers, Yukawa particles that belong to periodic arrays of crystallographic planes (111) are replaced by particles interacting via hard potential

(2) $$\beta u_{ij}=\left\{\begin{array}{cc} \infty , & r_{ij}<\sigma ,\\ 0, & r_{ij}\geq \sigma . \end{array}\right.$$

**Figure 1:**
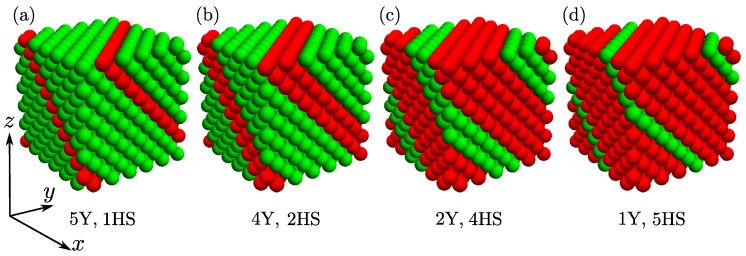
Typical structure of a studied crystal with nanolayers parallel to the (111) crystallographic plane. Yukawa’s particles are denoted with a green color and the hard spheres are marked with a red color. (**a**) a system with nanolayers consisting of a monolayer of hard spheres can be also seen as multilayer Yukawa system. This system is denoted in the text as 1HS or 6Y; (**b**) a system with nanolayers consisting of double layer of hard spheres; (**c**) a system with nanolayers consisting of four layers of hard spheres; (**d**) a system with nanolayers consisting of a monolayer of particles that interact via Yukawa potential. For more details, see Table 1.

The result is the system of Yukawa particles with nanolayers formed by hard spheres. Figure 1 illustrates a few examples of studied systems. From Figure 1a, one can see that the same system can be considered as the system with nanolayers consisting of monolayers of hard spheres or as the system with nanolayers consisting of multilayers of particles interacting through Yukawa potential. On the other hand, the system with nanolayers consisting of monolayers of particles interacting via Yukawa potential can be viewed as the system with nanolayers consisting of multilayers of hard spheres (see Figure 1c). Table 1 contains the details of the studied systems. The use of periodic boundary conditions in the directions x,y,z leads to the periodic structure with parallel nanolayers. Then, the systems described in the table can be considered as single super-cells. In order to analyse obtained results, we introduce *concentration* as a ratio of nanolayer particles ($N_{\mathrm{HS}}$) to all particles in the system (*N*) [66]

(3) $$c=\frac{N_{\mathrm{HS}}}{N}\times 100\%.$$

In order to determine the elastic properties of the studied systems, extensive Monte Carlo simulations in isobaric-isothermal ensemble (NpT) were performed using the Parrinello-Rahman method [40,67,68]. As the result of using that method, one obtains an elastic compliance 4th order tensor ($S_{ijkl}$) that describes elastic properties of studied systems entirely. Knowing each component of the compliance tensor, the Poisson’s ratio in arbitrary crystallographic direction and for any symmetry of the crystal can be determined using the formula [69]

(4) $$\nu_{nm}=-\frac{m_{i}m_{j}S_{ijkl}n_{k}n_{l}}{n_{p}n_{r}S_{prst}n_{s}n_{t}}\;,$$

where $\vec{\hat{n}}$ is a versor in the direction of the applied load and $\vec{\hat{m}}$ is the versor in the direction in which the Poisson’s ratio is measured, and the following relation has to be fulfilled: $\vec{\hat{n}}$·$\vec{\hat{m}}$=0. More details on calculating the Poisson’s ratio can be found in References [65,66].

Elastic properties of studied systems were determined for the following parameters of the Yukawa potential: κσ=10, βε=20, and pressure $p^{*}\equiv \beta P\sigma^{3}=100$ expressed in dimensionless units. The choice of these parameters was made on the basis of previous works [16,19,65]. The cut-off radius used in the simulations was equal to 2.5σ. The acceptance ratio of box moves and particle moves in the Monte Carlo method was 30%. In order to improve the accuracy of the obtained results, all physical values (elastic compliances and the Poisson’s ratios) were averaged over 10 independent runs for each of the studied systems. In other words, each system was simulated at least ten times. Single runs lasted $\left(2÷4\right)\times 10^{6}$ Monte Carlo (MC) cycles (depending on system sizes). The simulations of hard sphere system took $12\times 10^{6}$ MC cycles. First, $10^{6}$ cycles were treated as a period in which the system reaches the state of thermodynamic equilibrium.

## 3. Results and Discussion

Introducing a nanolayer into a perfect crystal with the face-centred cubic structure leads to change in the symmetry of the studied system. These changes were noticed in recent computer simulations [66]. A transition from fcc structure to the tetragonal one was observed. This is manifested by the change of the shape of simulation box from a cuboid to a rhombohedron. The matrix describing the simulation box of the system with nanolayers in the (111) plane has the form

(5) $$h=\left[\begin{array}{ccc} h_{xx} & h_{xy} & h_{xy}\\ h_{xy} & h_{xx} & h_{xy}\\ h_{xy} & h_{xy} & h_{xx} \end{array}\right]\;.$$

In this case, using Monte Carlo simulations utilizing the Parrinello–Rahman method, one obtains the following form the elastic compliance tensor (given in the matrix form in the Voigt notation [70]):

(6) $$S=\left[\begin{array}{cccccc} S_{11} & S_{12} & S_{12} & S_{14} & S_{15} & S_{15}\\ \cdot & S_{11} & S_{12} & S_{15} & S_{14} & S_{15}\\ \cdot & \cdot & S_{11} & S_{15} & S_{15} & S_{14}\\ \cdot & \cdot & \cdot & S_{44} & 0 & 0\\ \cdot & \cdot & \cdot & \cdot & S_{44} & 0\\ \cdot & \cdot & \cdot & \cdot & \cdot & S_{44} \end{array}\right].$$

The above form is the consequence of the symmetry of the $S_{ijkl}$ tensor. To determine the symmetry of the studied system uniquely, we bring the box matrix to its principal axes $h_{ij}^{\prime }=R_{ip}R_{jr}h_{pr}$ using the following rotation matrix

(7) $$R=\frac{1}{6}\left[\begin{array}{ccc} -3-\sqrt{3} & 3-\sqrt{3} & 2\sqrt{3}\\ 3-\sqrt{3} & -3-\sqrt{3} & 2\sqrt{3}\\ 2\sqrt{3} & 2\sqrt{3} & 2\sqrt{3} \end{array}\right]\;.$$

In the rotated coordinate system, the $h^{\prime }$ and $S^{\prime }$ matrices can be written, respectively, as

(8) $$h^{\prime }=\left[\begin{array}{ccc} h_{xx}^{\prime } & 0 & 0\\ 0 & h_{xx}^{\prime } & 0\\ 0 & 0 & h_{zz}^{\prime } \end{array}\right]\;,$$

(9) $$S^{\prime }=\left[\begin{array}{cccccc} S_{11}^{\prime } & S_{12}^{\prime } & S_{13}^{\prime } & S_{14}^{\prime } & -S_{15}^{\prime } & 0\\ \cdot & S_{11}^{\prime } & S_{13}^{\prime } & -S_{14}^{\prime } & S_{15}^{\prime } & 0\\ \cdot & \cdot & S_{33}^{\prime } & 0 & 0 & 0\\ \cdot & \cdot & \cdot & S_{44}^{\prime } & 0 & 2S_{15}^{\prime }\\ \cdot & \cdot & \cdot & \cdot & S_{44}^{\prime } & 2S_{14}^{\prime }\\ \cdot & \cdot & \cdot & \cdot & \cdot & 2(S_{11}^{\prime }-S_{12}^{\prime }) \end{array}\right]\;,$$

where

(10) $$\begin{array}{ccc} S_{11}^{\prime } & = & \frac{1}{2}\left(S_{11}+S_{12}-4S_{15}\right)+S_{44}, \end{array}$$

(11) $$\begin{array}{ccc} S_{12}^{\prime } & = & \frac{1}{6}\left(S_{11}+5S_{12}-2\left(4S_{14}+2S_{15}+S_{44}\right)\right), \end{array}$$

(12) $$\begin{array}{ccc} S_{13}^{\prime } & = & \frac{1}{3}\left(S_{11}+2S_{12}+S_{14}+2S_{15}-2S_{44}\right), \end{array}$$

(13) $$\begin{array}{ccc} S_{14}^{\prime } & = & \frac{1}{3}\left(S_{11}-S_{12}+S_{14}-S_{15}-2S_{44}\right), \end{array}$$

(14) $$\begin{array}{ccc} S_{44}^{\prime } & = & \frac{4}{3}\left(S_{11}-S_{12}-2S_{14}+2S_{15}+S_{44}\right). \end{array}$$

The above form of the $S^{\prime }$ matrix corresponds to the system of trigonal symmetry [70]. This clearly indicates that the systems with nanolayers in the (111) plane have trigonal symmetry.

**Figure 2:**
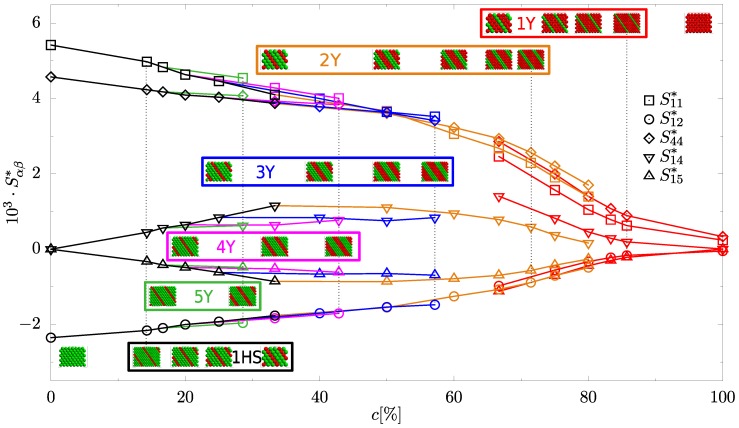
Elastic compliances versus concentration involved in particles of nanolayers in studied systems. Colors indicate the type of the system depending on the number of layers of appropriate particles in a nanolayer. 1HS (black) is the system with hard spheres monolayer. 1Y (red) is the system with monolayers of Yukawa particles. 2Y (light brown) is the system with double layers of Yukawa particles. 3Y (blue) is the system with triple layers of Yukawa particles. 4Y (magenta) is the system with four-layers of Yukawa particles. 5Y (green) is the system with five-layers of Yukawa particles. In addition, miniature images of studied systems are placed in the graph so that the centres of the structures coincided with the corresponding concentrations. The systems in the figure are grouped according to the numbers of layers in nanolayers. Miniatures of structures represent the studied systems seen from the $\left[\bar{1}10\right]$-direction. The color convention of data presentation from this figure is maintained in all following figures. Lines are drawn to guide the eyes.

Figure 2 shows the elements of elastic compliance matrix as a function of concentration for all studied systems. It can be seen that the values of elastic compliances ($S_{11}^{*},\;S_{12}^{*},\;S_{44}^{*}$) of systems in which nanolayers contain Yukawa multilayers (2Y–5Y) are, respectively, similar and the remaining elastic compliances ($S_{14}^{*},\;S_{15}^{*}$) present the same (continuous) trend as functions of concentration. However, at least in the concentration dependence of $S_{14}^{*}$, a discontinuity at the transition to the systems with Yukawa monolayers (1Y) is observed. For the latter systems, some components of elastic compliances ($S_{11}^{*},\;S_{14}^{*},\;S_{15}^{*}$) differ notably when compared with corresponding values of the same elements of double-layer Yukawa systems (2Y). This difference may be due to the lack of strong repulsion between the adjacent Yukawa layers inside the nanolayer in 1Y systems, which presents in multilayer systems (2Y–5Y). This has a significant effect on the auxetic properties of the 1Y systems.

**Figure 3:**
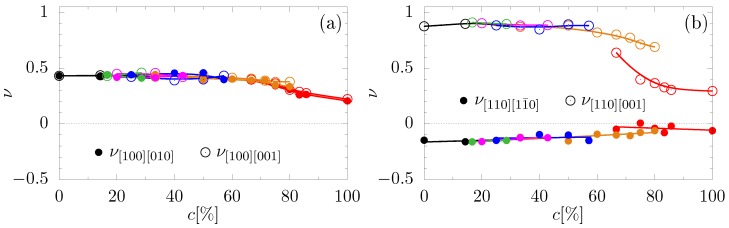

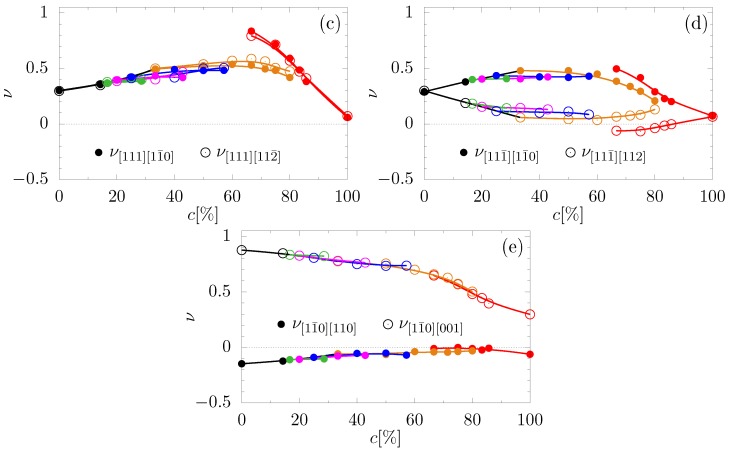
Poisson’s ratio in the main crystallographic directions versus concentration of particles of nanolayers in the studied system. The deformation of the system is applied respectively in the direction: (**a**) [100], (**b**) [110], (**c**) [111], (**d**) $\left[11\bar{1}\right]$, and (**e**) $\left[1\bar{1}0\right]$. The meaning of colors is the same as in Figure 2.

In Figure 3, the Poisson’s ratio in the main crystallographic directions has been shown. It can be observed here that the dependencies of Poisson’s ratio on *c* for Yukawa multilayer systems are very similar between each other. However, for certain crystallographic directions ([110][001], $\left[111\right]\left[1\bar{1}0\right]$, $\left[111\right]\left[11\bar{2}\right]$, $\left[11\bar{1}\right]\left[1\bar{1}0\right]$, $\left[11\bar{1}\right]\left[112\right]$), in the case of 1Y systems, we observe a qualitatively different character of the dependence of Poisson’s ratio from the concentration (*c*). Moreover, Poisson’s ratio in the $\left[11\bar{1}\right]\left[112\right]$ crystallographic direction exhibits negative values (see Figure 3d). This means that this direction is an auxetic one. It is worth noting that this happens while maintaining the auxeticity in the crystallographic direction of $\left[110\right]\left[1\bar{1}0\right]$ (see Figure 3b).

**Figure 4:**
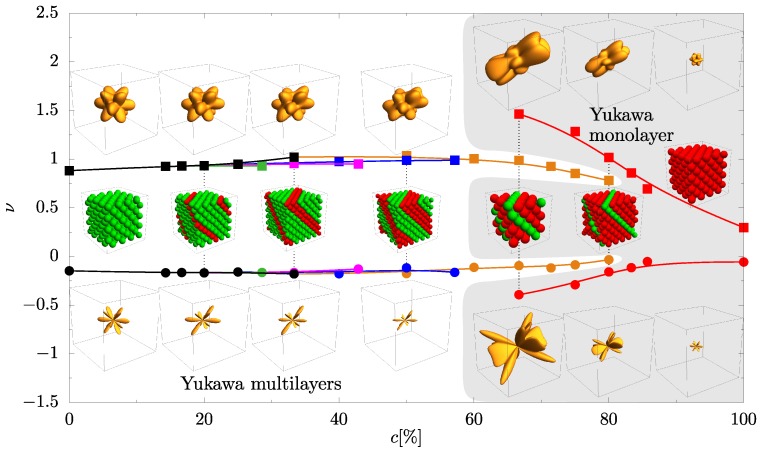
Maximal (squares) and minimal (circles) Poisson’s ratio taken from all crystallographic directions as a function of concentration of particles of nanoslit in studied system. All miniature images are placed in the graph so that the center of it coincided with the corresponding concentration (except pure Yukawa and HS systems). Upper row of miniature images correspond to the maximal values of Poisson’s ratio in all crystallographic directions. Lower row of miniature images describe the absolute value of minimal negative Poisson’s ratio in all crystallographic directions of studied systems. The studied structures of trigonal symmetry and crystallographic axes of three-dimensional plots have the same orientation as in Figure 1. The shaded area denotes the data on the graph concerned with the system with the nanolayer consisting of a monolayer of particles that interact via Yukawa potential. Lines are drawn to guide the eyes.

The dependencies of the minimum and maximum Poisson’s ratio values of the studied systems on the concentration are presented in Figure 4. Here, one can see a qualitatively different dependence of the Poisson’s ratio on the concentration in systems with Yukawa monolayer and systems with Yukawa multilayers. For multilayered Yukawa systems, the concentration weakly affects the extreme values of the Poisson’s ratio. Moreover, the extreme values of the Poisson’s ratio are barely dependent on the number of Yukawa layers in the nanolayers (see Figure 4). This is reflected in the results obtained for multilayered Yukawa systems, which are presented in Figure 4 in the range of concentrations from 0% to 80%. However, in the case of monolayers of Yukawa particles in the system, one can find in Figure 4 that the dependence of the Poisson’s ratio on the concentration is qualitatively different from those obtained for multilayered Yukawa systems. It is important to note that minimum value of Poisson’s ratio in the monolayer Yukawa system (1Y) at c=66.67% reaches the value −0.39(3) in the $\left[2\bar{1}2\right]\left[141\right]$ crystallographic direction.

In Figure 4 (see inserts), some structures studied for a few concentrations are presented. They illustrate extreme values of Poisson’s ratio in all crystallographic directions in the three-dimensional plot. If we assume that, in the plot, a vector from the origin of the coordinate system points to some point of three-dimensional surface, then a direction of the vector represents the direction of the applied stress ($\vec{\hat{n}}$), and its modulus has the extreme value of Poisson’s ratio in that direction. Thus, each point on the surface of the plot corresponds to the maximal value of Poisson’s ratio (top row of inserts in Figure 4) or absolute value of minimal negative Poisson’s ratio (bottom row of inserts in Figure 4) for the direction in which the stress is applied. This gives a qualitative picture of changes in the extreme values of Poisson’s ratio in all crystallographic directions due to the presence of nanolayers in the studied system. In the case of minimal Poisson’s ratio (Figure 4 bottom row), the increase in volume of the figure signals the enhancement of the auxetic properties in the system—this is observed for the Yukawa monolayers (shaded area in Figure 4). On the other hand, it can be seen that, for Yukawa multilayer systems, there are crystallographic directions in which the value of Poisson’s ratio changes with concentration (see Figure 3), although the extreme values of Poisson’s of the entire system, obtained from extremal values in all crystallographic directions, are almost constant.

Besides all this, it follows from Figure 4 that not only does the concentration of particles of nanolayer affect the elastic properties of the system, but also the distance between the layers in the nanolayer. In particular, an absence of inter-layers Yukawa interactions leads to sharp changes of the elastic properties of the system (see Figure 4). In order to quantitatively describe the change of the auxeticity in studied systems, recently, the degree of axeticity has been defined as [71]

(15) $$\chi =\sqrt[{3}]{\frac{3A}{4\pi }}\;,$$

where $A=\int_{0}^{2\pi }d\phi \int_{0}^{\pi }\sin\theta d\theta \int_{0}^{R(\theta ,\phi )}r^{2}dr$ is the integral of absolute values of mean negative Poisson’s ratios and $R\left(\theta ,\phi \right)=\frac{1}{2\pi }\int_{0}^{\pi }\left(\left|\nu_{n(\theta ,\phi )}\left(\alpha \right)\right|-\nu_{n(\theta ,\phi )}\left(\alpha \right)\right)d\alpha$ is the average over negative values of Poisson’s ratio in the direction of $\vec{\hat{n}}$. Here, the versor $\vec{\hat{n}}$ is determined in spherical coordinates. For more details, see Reference [71].

**Figure 5:**
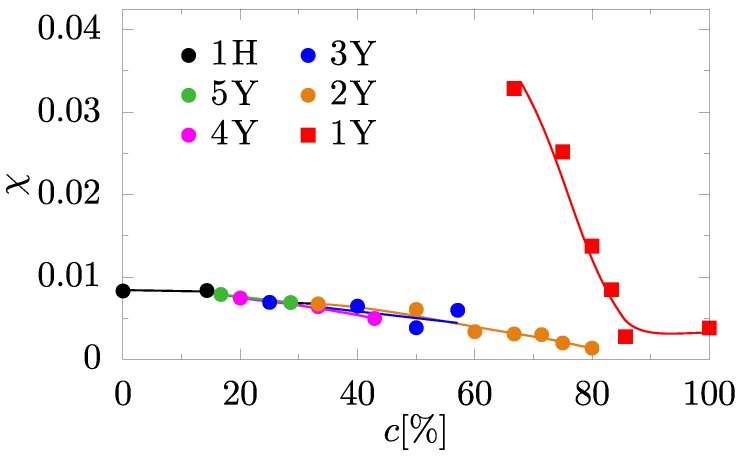
Degree of auxeticity as a function of concentration *c*. χ=0 corresponds to the case of non-auxetic system. Lines are drawn to guide the eyes.

In Figure 5, the degree of auxeticity of studied systems as a function of concentration is presented. A qualitative difference in elastic properties of monolayered Yukawa systems and multilayered Yukawa systems is observed. In the case of monolayered Yukawa systems, an essential enhancement of auxeticity was found. However, the increase of concentration of nanolayer particles is causing a weakening of auxetic properties of the system.

## 4. Conclusions

This work is a continuation of the study on the effect of structural modifications on elastic properties, and particularly on the auxetic properties of the model of nanocomposites. Comprehensive Monte Carlo simulations revealed that the structural modification of the Yukawa system in the form of nanolayers in the (111) crystallographic plane can lead both to strengthening as well as weakening the auxetic properties of the system. Reduction of auxetic properties is achieved by increasing the concentration of nanolayer particles in multilayer Yukawa systems, whereas monolayered Yukawa systems exhibit the enhancement of auxetic properties. The latter is the appearance of a new auxetic direction ($\left[11\bar{1}\right]\left[112\right]$) while preserving other, already existing, auxetic directions in the system.

The extreme values of the Poisson’s ratio of the studied systems were also determined. The presentation of them in the form of a three-dimensional plot is suitable for a quick qualitative assessment of changes in the auxetic properties of the whole system caused by the modification of the structure of the studied system. On the other hand, a use of degree of auxeticity gives a quantitative description of auxetic properties of the system under study.

The results presented in this paper demonstrate another (simple and efficient) way of controlling the elastic properties of materials and show the consequences of introducing the nanolayers in the (111) crystallographic plane of given crystal. This information may be useful for the construction of nanocomposites, and may indicate directions for further research on materials with desired elastic properties.

## Figures and Tables

**Table 1:** Examples of structural modifications of studied systems. Each structure was based on the face-centered cubic (fcc) lattice which unit cell contains four atoms. *n* is the number of unit cells in direction’s x,y,z. $N=4n^{3}$ is the number of particles in the system. $N_{\mathrm{HS}}$ is the number of particles in nanolayers. *c* is the concentration of nanolayers particles in the system. ρ=N/V is the density of the system. $n_{Y}$ is the number of layers of Yukawa particles in a nanolayer. $n_{\mathrm{HS}}$ is the number of layers of hard spheres in a nanolayer. The description column holds the abbreviations that indicate the number of layers in a nanolayer, and are used in figures and in the text to refer to those systems.

*n*	*N*	$N_{\mathrm{HS}}$	*c* [%]	ρ	$n_{Y}$	$n_{\mathrm{HS}}$	Description
7	1372	196	14.29	0.9940(4)	6	1	6Y,1HS
6	864	144	16.67	1.0042(9)	5	1	5Y,1HS
7	1372	392	28.57	1.0358(7)	5	2	5Y,2HS
5	500	100	20.00	1.0190(7)	4	1	4Y,1HS
6	864	288	33.33	1.0554(4)	4	2	4Y,2HS
7	1372	588	42.86	1.0854(9)	4	3	4Y,3HS
4	256	64	25.00	1.0422(2)	3	1	3Y,1HS
5	500	200	40.00	1.0848(1)	3	2	3Y,2HS
6	864	432	50.00	1.1189(3)	3	3	3Y,3HS
7	1372	784	57.14	1.1472(8)	3	4	3Y,4HS
3	108	36	33.33	1.0840(4)	2	1	2Y,1HS
4	256	128	50.00	1.1343(2)	2	2	2Y,2HS
5	500	300	60.00	1.1729(8)	2	3	2Y,3HS
6	864	576	66.67	1.2036(3)	2	4	2Y,4HS
7	1372	980	71.43	1.2281(8)	2	5	2Y,5HS
8	2048	1536	75.00	1.2476(1)	2	6	2Y,6HS
10	4000	3200	80.00	1.2753(5)	2	8	2Y,8HS
3	108	72	66.67	1.2406(4)	1	2	1Y,2HS
4	256	192	75.00	1.2789(1)	1	3	1Y,3HS
5	500	400	80.00	1.3021(1)	1	4	1Y,4HS
6	864	720	83.33	1.3159(9)	1	5	1Y,5HS
7	1372	1176	85.71	1.3247(4)	1	6	1Y,6HS

## Abbreviations

The following abbreviations are used in this manuscript:

HCRYP	hard-core repulsive Yukawa potential
HS	hard spheres potential
